# Impact of Walking and Running on the Heel bone: the Adventist Health Study-2

**DOI:** 10.9734/JSRR/2015/17962

**Published:** 2015-04-30

**Authors:** Vichuda Lousuebsakul-Matthews, Donna Thorpe, Raymond Knutsen, W. Larry Beeson, Gary E. Fraser, Synnove F. Knutsen

**Affiliations:** 1 Center for Nutrition, Healthy Lifestyle and Disease Prevention, School of Public Health, Loma Linda University, Loma Linda, CA, USA.; 2 Los Angeles County, Department of Health Services, Los Angeles County, Los Angeles, CA, USA.; 3 Department of Physical Therapy, School of Allied Health Professions, Loma Linda University, Loma Linda, CA, USA.

**Keywords:** Physical activity, musculoskeletal health, exercise, broadband ultrasound attenuation

## Abstract

**Aims:**

Physical activity is well recognized for its bone health benefit. We examined the benefit of walk/run/jog on bone health using broadband ultrasound attenuation (BUA) of the calcaneus.

**Methodology:**

Caucasian and African American males (n=593) and females (n=1,106) had their calcaneal BUA measured two years later after enrollment into the AHS-2. The association between calcaneal BUA (dB/Mhz) and the distance of walk/run/ jog level per week (miles) was assessed using multiple linear regression.

**Results:**

In a multivariable model adjusted for important covariates, BUA was positively associated with BMI (*P* < .001), total calcium intake (*P* =0.31), total protein intake (*P* =0.38) and inversely associated with age (*P* < .001) and smoking (*P* < .05). Compared to women who did not walk/ run/ jog, women walking 10 or more miles per week had an increase in BUA by 4.08 (dB/Mhz) (*P*_trend_=0.03). Similarly, compared to men who did not walk/ run/ jog, men walking 10 or more miles per week had an increase in BUA by 5.97 (dB/Mhz) (*P*_trend_=0.01).

**Conclusions:**

We concluded that BUA is positively associated with walk/ run/jog after accounting for age, BMI, smoking status, calcium intake, protein intake and estrogen usage.

## 1. INTRODUCTION

The transmission of ultrasound waves through bone matrix via scattering and absorption yields broadband ultrasound attenuation (BUA). Attenuation of ultrasound waves depends upon the bone’s microstructure, elasticity, anisotropy and mineral density [1]. BUA is widely used to assess bone health as it is a cost-effective and convenient screening method with lack of ionizing radiation. BUA has been found to have high correlation with bone mineral density (BMD) measured by dual energy *x-*ray absorptiometry (DXA) [2–5]. It is also able to predict the risk of bone fracture to a similar degree as DXA [6–11].

Previous animal studies have shown some evidence that the mechanical loading stimulus occurring during weight-bearing exercise stimulates the bone remodeling process [12]. Several studies suggest that the trabecular structure of the heel bone is directly impacted by the heel striking motion during exercise such as walking or running [13–15]. Since the heel bone consists mainly of cancellous bone, the type that is mostly affected by osteoporotic fractures, changes in heel bone structure may be an indication of bone health. Taken together, BUA of the heel bone may therefore be an accurate reflection of an individual’s habitual physical activity pattern. Previous studies have shown that there is an independent effect of physical activity on BUA even when adjusting for BMD [2,11,16]. The adaptive response of bone to physical activity was reported in a recent meta-analysis showing a significant improvement in calcaneal BUA (0.98 standardized mean difference, (p< 0.0001) among individuals engaging in exercise intervention of 4 to 36 months compared to the control groups [17].

In this study, we ascertained the effect of walking/ running/ jogging on bone health using calcaneal BUA among Caucasian and African American males and females adjusting for demographic and lifestyle factors (smoking, protein intake, etc).

## 2. MATERIALS AND METHODS

### 2.1 Study Population

Subjects were enrollees in the Adventist Health Study 2 (AHS-2), a large National Cancer Institute (NCI) funded cohort study investigating the relationship between lifestyle factors and several disease outcomes. The study has been described in detail elsewhere [18] and consists of Adventists over 30 years of age throughout the United States and Canada who completed a comprehensive lifestyle and dietary questionnaire at enrollment between 2002 and 2007. This study was approved by the Loma Linda University Institutional Review Board (IRB).

One thousand and eleven subjects were randomly selected through a 2 stage sampling process to participate in a large calibration study. In addition, a sample of 1,119 subjects from the AHS-2 attended the pilot church clinics that were conducted in preparation for a nationwide clinic study. Of these study subjects, there were 2,037 Caucasian and African American males and females, who attended clinics arranged in conjunction with the AHS-2. We excluded those with a history of minor accidental fracture (n=260), those with missing data on physical activities (n=47) and BUA measurement (n=31), leaving a total of 1,699 Caucasian and African American males and females for the analysis.

### 2.2 Physical Activity Questionnaire

Physical activity was assessed using two questionnaire items focusing on subject’s physical activity during the last twelve months. Questions were part of a large self-reported baseline lifestyle questionnaire completed at enrollment into the AHS-2. The questions captured the distance and the frequency per week that the subject engaged in weight-bearing exercises such as walking, running or jogging. The question on the frequency of exercise was “How many of these “walk” or “run” or “jog” workouts do you usually do per week?” The response choices were: “less than once/week”, “1 time per week”, “2 times per week”, “3 times per week”, “4 times per week”, “5 times per week”, “6 or more times per week”. We assigned a frequency score of 0 to 6, respectively, corresponding to the choices given. The question on the distance of exercise was “How many miles do you average per “walk” or “run” or “jog” workout?” The responded choices were: “1/4 mile or less”, “1/2 mile”, “1 mile”, “1 ½ miles”, “2 miles”, “3 miles” and “4 or more miles”. We assigned a distance score of 0.25, 0.5, 1, 1.5, 2, 3 and 4 miles, respectively, corresponding to the choices given (*e.g*., subject who walked 2.5 miles would receive a score of 2 since it reflected a walking distance between 2 to 3 miles). An algorithm was developed based on the multiplication of the frequency score and the distance score and further categorized into six physical activity distance levels (0 mile, 0.1–2.5 miles, 2.6 – 5 miles, 5.1 – 7.5 miles, 7.6 – 10 miles, > 10 miles) per week.

### 2.3 Food Frequency Questionnaire (FFQ)

Dietary information was collected as part of enrollment into the parent AHS-2 study using a comprehensive self-administered and validated food frequency questionnaire (FFQ) reporting on the subject’s dietary intake during the last twelve months. Subjects were asked to report how frequently they consumed a food: “never”, “1–3 times per month”, “1 time per week”, “2–4 times per week”, “5–6 times per week”, “1 per day”, and “2 or more times per day”. In addition, subjects were given the size of an average serving of that particular food and asked to mark if they consumed the average size, half this size or one-and-a-half or larger of the average size. Based on this information from the FFQ, several nutrient indices (total protein (g) total calcium (mg)) were developed.

### 2.4 Lifestyle Questionnaire

At enrollment, in addition to the FFQ and exercise information, participants completed questions on medical history, smoking and anthropometrics. There was also a female section which included menopausal status and estrogen usage. The question on fractures was asked “How many of your fractures (since the age of 35) were due to minor accidents (falling from standing height or less, tripping over an object, falling from one step, etc.)?” Those who reported one or more fractures were excluded from our study population (n=254).

### 2.5 Clinic Visit and Bone Ultrasound Measurement

Approximately two years after enrollment into the parent study, subjects were invited to attend a clinic either as part of the calibration study [19] or as part of the church clinic pilot study [20]. During the clinics, anthropometrics were measured, broadband ultrasound attenuation (BUA) was assessed at the site of the calcaneus using the contact bone ultrasound analyzer (CUBA) system [21]. A calibration check was performed daily before any measurement was performed on that day. Ultrasound gel was applied to both sides of the calcaneus of the dominant foot before the subject placed the heel on a foot rest, cradling the calcaneus between the two opposing ultrasound transducers which measured the density and structure of the calcaneus. All subjects had their BUA measurement done by the same CUBA system and by trained technicians.

### 2.6 Statistical Analysis

Chi-Square tests were used to determine the association between physical activity level per week and selected predictor variables (Table 1). Multiple linear regression was used to determine the association between exercise level and BUA adjusted for nutrient and demographic variables. A basic model, with BUA as the dependent variable, was developed with age, gender, race, BMI and physical activity distance level in miles per week (0 mile, 0.1–2.5 miles, 2.6 – 5 miles, 5.1 – 7.5 miles, 7.6 – 10 miles, > 10 miles of walk/run/ jog). A multivariate model further adjusted for smoking status, total calcium intake (< 1000 mg (median) vs. >= 1000 mg), total protein intake (< 60 g (median) vs. >= 60 g), menopausal status and estrogen usage (only females). In order to examine the effect of estrogen use and menopausal status together on BUA, we categorized the menopausal status and estrogen usage into four categories: postmenopausal with current estrogen use, postmenopausal without current estrogen use, premenopausal with current estrogen use and premenopausal without current estrogen use. Postmenopausal women, who were not current estrogen users, were in a reference group in a multivariate model. Gender specific analysis was performed in both basic and multivariate models (Table 3 & 4). Sensitivity analysis was performed by excluding subjects with history of osteoporosis (n=113) in a gender specific analysis. All statistical analyses were performed using SAS 9.3 (SAS Institute, Cary, NC).

## 3. RESULTS

### 3.1 Univariate Analyses

Approximately 27% (n=465) of our participants reported the lowest level of physical activity (0 mile/wk) whereas 12% (n=203) reported walking or running more than 10 miles per week. No significant association was observed between physical activity level and BUA (*P*=0.08), however the significant association was observed in the internal comparison between those whose walk/ run/ jog was greater than 10 miles to those with lower level of physical activity (*P* < .05). There was no significant difference in the mean age of participants or BUA by the level of walking or running. However, there was a significant difference in BMI by the level of physical activity. Those who reported engaging in longer distance of walking or running had lower BMI compared to those whose weekly walk was 2.5 miles or less (Table 1). Out of 1,106 females, 10% (n=114) reported walking or running more than 10 miles per week whereas 15% (n=89) of males reported walking/running in the same capacity. A higher proportion (14%) of 929 Caucasians reported walking or running more than 10 miles per week compared to 10% among the Blacks (n=77). There was a significant association between the level of daily calcium intake and physical activity level. Men and women with lower calcium intake (< 1000 mg per day) were more likely to walk/ run/ jog less than 2.6 miles per week compared to those with higher calcium intake. Similarly, a higher proportion of those with higher protein intake (60+ gm per day) reported walking/ running more miles per week than those with lower protein intakes. No significant association was found between smoking status and physical activity level. No significant association was observed between physical activity level and estrogen usage among females.

In a basic regression model adjusting for age, race and gender, walking/ running/ jogging at level 5 ( 7.6 – 10 miles/wk) and level 6 (>= 10 miles/ wk) significantly increased BUA by 4.26 dB/Mhz and 5.83 dB/Mhz, respectively, compared to the lowest level (0 mile/wk) (Table 2). BUA was significantly higher in males compared to females, in Blacks compared to Whites and in those with higher BMI. As expected, age was inversely associated with BUA with a decrease of 0.56 dB/Mhz per year. No interaction effect was found between race,BUA and physical activity level when tested in the basic model. In a race-specific model, walking/ running/ jogging at level 6 (>= 10 miles/ wk) significantly increased BUA among Whites and Blacks by 6.06 dB/Mhz and 5.56 dB/Mhz, respectively (data not shown).

### 3.2 Multivariable Analyses

When further adjusting for smoking status, total calcium intake and total protein intake, the effect estimates for the variables in the basic model remained virtually unchanged and the associations of walk/run/jog with BUA was stable with an increase of 3.9 dB/Mhz and 5.64 dB/Mhz, respectively, for walk/ run/ jog at level 5 (7.6 – 10 miles/wk) and level 6 (> 10 miles/ wk) (Table 2). A trend test was statistically significant (*P* <0.001). BUA was negatively associated with a positive smoking history and positively associated with total daily calcium intake and protein intake, although these associations were not statistically significant.

The association between walk/ run/ jog and BUA, comparing level 6 with level 1, was slightly stronger in males with a 5.97 dB/Mhz increase compared to 4.08 dB/Mhz in females (after also adjusting for current estrogen use) (Tables 3 and 4). The negative association of age with BUA was stronger in females than males, 0.65 dB/Mhz and 0.35 dB/Mhz, respectively, for each year of age (Table 3 & 4). For each increasing unit of BMI (kg/m^2^), BUA significantly increased by 0.35 dB/Mhz and 0.57 dB/Mhz among males and females, respectively (Table 3 & 4).

BUA was significantly higher among Blacks compared to Whites overall, and for both sexes separately, though much more so among females (1.76 dB/Mhz and 5.00 dB/Mhz among males and females, respectively) (Table 3 & 4). Among post-menopausal females, current estrogen users had 10.14 dB/Mhz higher BUA than past/ never users. Compared to post-menopausal women who reported currently not using estrogen, BUA significantly increased by 14.0 dB/Mhz and 3.1 dB/Mhz among premenopausal women who were current estrogen users and past/never users, respectively (Table 4). In a sensitivity analysis excluding subjects with history of osteoporosis, the association between walk/ run/ jog and BUA remained significant at level 6 compared to level 1 (data not shown).

## 4. DISCUSSION

In our study, walking/ jogging more than 10 miles per week significantly increased calcaneus BUA by 4.08 dB/Mhz and 5.97 dB/Mhz among women and men, respectively. Our findings are in agreement with previous studies examining the effect of physical activity on calcaneal BUA in similar adult populations. Among postmenopausal women, a brisk walking of approximately 20 minutes per day for a period of 12-months can significantly improve calcaneal BUA by 3 dB/Mhz compared to control subjects [22]. In the following year, 17 control subjects who took on brisk walking for an average of 20 minutes per day, had calcaneal BUA increased significantly by 7.4% or approximately 4.8 dB/Mhz [22]. In a study of elderly Japanese women, walking 8000 steps per day on average significantly increased calcaneal BUA by 8 dB/Mhz compared to walking zero steps per day [23]. Ay A et al. exposed postmenopausal women to aquatic exercise or weight-bearing exercise for 6 months and found significantly increased calcaneal BUA by 1.1 dB/Mhz and 1.6 dB/Mhz, respectively [24]. In a 12-month exercise intervention among postmenopausal Caucasian women, there was a non-significant improvement in calcaneal BUA among women who participated in line dancing and line dancing plus squats by 0.43 dB/Mhz and 2.3 dB/Mhz, respectively [25]. In a study by Kastelan et al, all ultrasound parameters were significantly higher in physical education students as compared to medical students (*P* < 0.001). The multiple regression model of the quantitative ultrasound index confirmed that the type of academic program students attended was the single most significant predictor variable in both genders [26].

Most previous studies among males consisted mainly examined younger men [27–29]. Daly et al studied the effect of 18-month gymnastics training among young male gymnasts, and found that calcaneal BUA significantly increased by 4.9 dB/Mhz (12.8%) in the intervention group but not in the control group [27]. Babaroutsi reported that 26–33 year old Greek males, who engaged in non-supervised physical activity, had a significantly higher quantitative ultrasound (QUS) index compared to those who did not [28]. In a study examining the effect of ten weeks military training, Etherington et al found a 10% difference in BUA (*P* <.05) in men belonging to the highest quartiles of the exercise index as compared to those in the lowest [29]. Despite differences in BUA measurement devices, and differences in the type, intensity and duration of exercise, studies have shown an overall positive association between physical activity and calcaneal BUA among adults, as reported in a recent meta-analysis by Babatunde et al. [17].

It is well established that osteogenic stimulus requires mechanical loading on bone and that the relationship between the loading force and the osteogenic response is strongly linear within stress tolerance limits. Thus, high impact weight bearing exercise such as jumping has been found to produce the strongest osteogenic response [30,31]. Despite the fact that walking, jogging or running are not high impact weight bearing exercises, we observed in our study a positive effect of this type of exercise on bone health assessed by BUA. However, the observed effect of physical activity on BUA may, at least in part, be independent of any concomitant change in BMD. This has been suggested by contrasting outcomes of studies comparing bone health in swimmers and sedentary controls [32–34]. In studies using quantitative ultrasound (QUS) swimmers showed higher QUS indices than controls whereas in studies measuring BMD no differences have been found. Several authors suggest that BUA is influenced by the microstructure of the bone, which cannot be detected by DXA [34,35].

In our study, the benefit of walking or jogging more than 10 miles per week on calcaneal BUA was greater among men compared to women. A gender difference in the effect of exercise on BUA has previously been reported for adolescent boys and girls.

In a cross-sectional study of Japanese students, 15 – 20 years of age, calcaneal BUA was significantly higher in the exercise group compared to non-exercise group by 8.7 dB/Mhz and 4.6 dB/Mhz in males and females, respectively [36]. In an 8-month follow-up study by Weeks et al, ten minutes jumping twice a week increased calcaneal BUA by 3.8 dB/Mhz and 1.8 dB/Mhz among adolescent boys and girls, respectively [37] and was only significant among boys. In adults, the observed differences between males and females may have been due to gender differences in rate of bone loss with aging. Compared to men, the effect of age-related decline in BUA has been shown to be five times greater in women [38,39]. Drysdale et al studied the effect of exercise on BUA in marathon runners and non-runners, and reported a delay in the rate of decline of BUA per year of −0.35 to −0.25 dB/MHz for male runners vs non-runner, and of −0.51 to −0.15 dB/MHz for females runners vs non-runners [40]. Brunner et al studied older Germans and found that each additional year of age significantly decreased calcaneal BUA by 0.38 dB/MHz and 0.14 dB/MHz in females and males, respectively [41]. Furthermore, in the same age adjusted multivariate model, calcaneal BUA explained a greater portion of BUA variation in women than in men. In our study, using an identical basic model, the variability of calcaneal BUA was better explained by age, BMI and physical activity among females as compared to males (28% vs. 8%). This is in agreement with findings among elderly Japanese women where age, BMI and walking activity explained 24.6% of the variance of calcaneal BUA [23]. In the EPIC-Norfolk study, age, weight and height accounted for 27% and 3% of the variance of calcaneal BUA among women and men, respectively [39]. This finding indicated that the effect of age on BUA was greater among females compared to males.

We found that, being Black was associated with greater BUA (by 5.00 dB/MHz and 1.76 dB/MHz, among females and males, respectively), when compared to White subjects. Thus, the effect of race was more pronounced among females compared to males. The observed difference in the effect of race may have been due to a higher peak bone mass achieved in the young adult years among Blacks compared to Whites. In our study, younger Black females (<=40 years) and younger Black males (<=40 years) had a significantly higher calcaneal BUA compared to their White counterparts by 7.04 dB/MHz and 6.91 dB/MHz, respectively (data not shown). Aloia et al found that among white and black women of the same height and weight, Black women have both a higher skeletal mass and lean mass [42]. Many authors have suggested that even though the rate of bone loss is similar in both Black and White women, Black women [43] in general have a higher peak bone mass in the younger years [44–46]. Black women also have a higher level of testosterone, which tends to increase bone density [42,47].

Our findings of the effects of age and BMI are similar to that reported by others [39,48,49]. Estrogen usage as well as protein and calcium intake were beneficial to bone as seen in BUA in our study, and this is also shown by others [50–52]. Furthermore, we found that smoking was detrimental to bone health, and this has been observed by others as well [53–54].

## 5. CONCLUSION

Our findings suggest that walking, running or jogging can in addition improve calcaneal BUA in both males and females.

### 5.1 Strengths and Limitations

The strength of our study is the employment of our validated physical activity questionnaire [55,56]. A limitation is that there might have been a change in the physical activity pattern during the 2- year period between the baseline questionnaire and the calcaneal BUA measurement. The benefits of physical activity among middle aged and elderly individuals has been shown to reduce the rate of bone loss due to aging and improvement in balance, leg strength, flexibility and endurance, which has the potential to reduce the risk of falls and fractures [57]. Recommendations to walk or run should be part of a non-pharmacologic intervention to optimize bone health and prevent osteoporosis.

## Figures and Tables

**Table 1.** Demographic and Lifestyle characteristics among 1,699 Caucasian and African-American Males and Females by Physical Activity Level

Walk/ Run/ Jog						
	Level 1 (0 mile/wk) (n=465)	Level 2 (0.1–2.5 miles/wk) (n=391)	Level 3 (2.6–5 miles/wk) (n=259)	Level 4 (5.1–7.5 miles/wk) (n=192)	Level 5 (7.6–10 miles/wk) (n=189)	Level 6 (10+ miles/wk) (n=203)
**BUA** (dB/MHz)(Mean, SD)	80.2(21.3)	80.9(20.7)	79.8(19.5)	80.2(22.1)	82.6(20.5)	84.9(21.6) *P*^1^ =*0.08*
**Age** (Mean, SD)	58(14)	57(14)	58(14)	59(14)	58(12)	59(12) *P*^1^*=0.60*
**BMI** (kg/m^2^) (Mean, SD)	28.6 (7.9)	28.0(7.1)	26.9 (6.3)	26.4(6.8)	26.5 (5.8)	26.5 (5.0) *P*^1^ *<0.001*
**Gender:**						
Females	61% (n=284)	70% (n=272)	67% (n=173)	70% (n=135)	68% (n=128)	56% (n=114)
Males	39% (n=181)	30% (n=119)	33% (n=86)	30%(n=57)	32% (n=61)	44% (n=89)
**Race:**						
Whites	49%(n=229)	52%(n=202)	54%(n=141)	60%(n=116)	61%	62%
Blacks	51%(n=236)	48%(n=189)	46%(n=118)	40%(n=76)	39%(n=74)	38%(n=77) *P*^1^*=0.01*
**Smoking Status:**						
Never smokers	83%(n=386)	85% (n=331)	87% (n=226)	87%(n=167)	90% (n=170)	83% (n=168)
Ever smokers	17%(n=79)	15%(n=60)	13% (n=33)	13%(n=25)	10% (n=19)	17%(n=35) *P*^1^*=0.18*
**Total Daily Calcium (mg):**						
< 1000	59%(n=276)	56%(n=218)	47%(n=121)	42%(n=80)	42%(n=80)	41%(n=84)
≥ 1000	41%(n=189)	44%(n=173)	53%(n=138)	58%(n=112)	58%(n=109)	59%(n=119) *P*^1^ *<0.001*
**Daily Dietary Protein (gm):**						
< 60	53%(n=246)	47%(n=185)	51%(n=131)	49%(n=94)	39%(n=74)	41%(n=84)
≥60	47%(n=219)	53%(n=206)	49%(n=128)	51%(n=98)	**61%(n=115)**	59%(n=119) *P*^1^=*0.01*
**Menopausal / Estrogen use:**						
Postmenopausal with no current estrogen usage	55%(n=155)	44%(n=119)	40%(n=70)	45%(n=61)	48%(n=62)	49%(n=56)
Postmenopausal with current estrogen usage	9%(n=26)	13%(n=35)	14% (n=25)	13%(n=18)	15%(n=19)	11% (n=13)
Pre menopausal with no current estrogen usage	35% (n=99)	42% (n=113)	42% (n=73)	41% (n=55)	34% (n=43)	37% (n=42)
Premenopausal with current estrogen usage	1%(n=4)	2%(n=5)	3%(n=5)	1%(n=1)	3%(n=4)	3%(n=3) *P*^1^*=0.36*

1 Chi-Square/ ANOVA

**Table 2.** Associations between Distance (miles) of Walk/ Run/ Jog and BUA (dB/MHz) Among 1,699 Caucasian and African-American Males and Females - Multiple Linear Regression Model

	Basic Model^1^		Multivariate Model^2^	
	Parameter Estimate	*P-* Value	Parameter Estimate	*P-* Value
**Age** *(Continuous)*	−0.56	<0.001	−0.56	<0.001
**BMI (kg/m**^**2**^**)** *(Continuous)*	0.51	<0.001	0.53	<0.001
**Gender:**				
Females	Referent		Referent	
Male	11.17	<0.001	11.42	<0.001
**Race:**				
Whites	Referent		Referent	
Blacks	3.02	<0.05	3.36	<0.05
**Walk/ Run/Jog** (per week):				
Level 1 (0 mile)	Referent		Referent	
Level 2 (0.1–2.5 miles)	1.19	0.35	1.11	0.38
Level 3 (2.6 −5 miles)	1.43	0.32	1.26	0.38
Level 4 (5.1 −7.5 miles)	2.66	0.09	2.45	0.12
Level 5 (7.6–10 miles)	4.26	0.01	3.90	0.02
Level 6 (10 + miles)	5.83	0.001	5.64	<0.05
	***Trend P* <0.001**		***Trend P* <0.001**	
**Smoking Status:**				
Never smokers	n/a		Referent	
Ever smokers	n/a		−2.55	0.05
**Total Calcium (mg):**				
< 1000	n/a		Referent	
≥ 1000	n/a		1.02	0.31
**Dietary Protein (g):**				
< 60	n/a		Referent	
≥60	n/a		0.86	0.38

1 Adjusted for age, race, gender, BMI (R Square =24%; Adjusted R Square =24% )

2 Adjusted for age, race, gender, BMI, smoking status, dietary protein, dietary calcium (R Square =24%; Adjusted R Square =24%)

**Table 3.** Associations between Distance (miles) of Walk/ Run/ Jog and BUA (dB/MHz) Among 593 Caucasian and African-American Males - Multiple Linear Regression Model

	Basic Model^1^		Multivariate Model^2^	
	Parameter Estimate	*P-* Value	Parameter Estimate	*P-* Value
**Age** *(Continuous)*	−0.38	<0.001	−0.35	<0.001
**BMI (kg/m**^**2**^**)** *(Continuous)*	0.30	0.04	0.35	0.02
**Race:**				
Whites	Referent		Referent	
Blacks	0.12	0.94	1.76	0.32
**Walk/ Run/Jog** (per week):				
Level 1 (0 mile)	Referent		Referent	
Level 2 (0.1–2.5 miles)	1.64	0.47	0.67	0.77
Level 3 (2.6–5 miles)	0.30	0.91	−0.21	0.93
Level 4 (5.1 −7.5 miles)	6.02	0.04	5.01	0.09
Level 5 (7.6–10 miles)	5.26	0.07	4.22	0.14
Level 6 (10 + miles)	6.66	0.01	5.97	0.02
	***Trend P< 0.05***		***Trend P=0.01***	
**Smoking Status:**				
Never smokers	n/a		Referent	
Ever smokers	n/a		−6.62	*P< 0.05*
**Total Calcium (mg):**				
< 1000	n/a		Referent	
≥1000	n/a		1.78	0.33
**Dietary Protein (g):**				
< 60	n/a		Referent	
≥60	n/a		2.00	0.25

1 Adjusted for age, race, BMI (R Square =9%; Adjusted R Square =8%)

2 Adjusted for age, race, BMI, smoking status, dietary protein, dietary calcium (R Square =11%; Adjusted R Square =10%)

**Table 4.** Associations between Distance (miles) of Walk/ Run/ Jog and BUA (dB/MHz) Among 1,106 Caucasian and African-American Females - Multiple Linear Regression Model

	Basic Model^1^		Multivariate Model^2^	
	Parameter Estimate	*P*- Value	Parameter Estimate	*P*- Value
**Age** (*Continuous)*	−0.67	<0.001	−0.65	<0.001
**BMI (kg/m**^**2**^**)** *(Continuous)*	0.55	<0.001	0.57	<0.001
**Race:**				
Whites	Referent		Referent	
Blacks	4.36	<0.001	5.00	<0.001
**Walk/ Run/ Jog** (per week):				
Level 1 (0 mile)	Referent		Referent	
Level 2 (0.1–2.5 miles )	0.87	0.56	0.37	0.80
Level 3 ( 2.6 – 5 miles)	1.27	0.46	0.42	0.80
Level 4 ( 5.1 – 7.5 miles)	0.69	0.71	0.39	0.83
Level 5 ( 7.6 – 10 miles)	3.39	0.07	2.75	0.14
Level 6 ( 10 + miles)	4.40	0.03	4.08	0.04
	***Trend P< 0.05***		***Trend P=0.03***	
**Smoking Status:**				
Never smokers	n/a		Referent	
Ever smokers	n/a		−0.39	0.80
**Total Calcium (mg):**				
< 1000	n/a		Referent	
≥ 1000	n/a		0.23	0.84
**Dietary Protein (g):**				
< 60	n/a		Referent	
≥ 60	n/a		0.36	0.75
**Menopausal / Estrogen use:**				
Postmenopausal with no current estrogen usage	n/a		Referent	
Postmenopausal with current estrogen usage	n/a		10.14	<0.001
Premenopausal with no current estrogen usage	n/a		3.07	0.02
Premenopausal with current estrogen usage	n/a		14.02	<0.001

1 Adjusted for age, race, BMI (R Square =28%; Adjusted R Square =28%)

2 Adjusted for age, race, BMI, smoking status, dietary protein, dietary calcium, menopausal status and estrogen usage ( R Square =31.5%; Adjusted R Square =30.6%)
